# Differential impact on motor unit characteristics across severities of adult spinal muscular atrophy

**DOI:** 10.1002/acn3.51906

**Published:** 2023-09-21

**Authors:** Kristina Marie Kelly, Jordan Mizell, Ladan Bigdeli, Samuel Paul, Marco Antonio Tellez, Amy Bartlett, Sarah Heintzman, Jerold Everett Reynolds, Gary Brent Sterling, Kiran Francis Rajneesh, Stephen James Kolb, Bakri Elsheikh, William David Arnold

**Affiliations:** ^1^ Department of Physical Medicine & Rehabilitation University of Missouri Columbia MO USA; ^2^ NextGen Precision Health University of Missouri Columbia MO USA; ^3^ College of Medicine The Ohio State University Columbus OH USA; ^4^ Department of Neurology The Ohio State University Wexner Medical Center Columbus OH USA; ^5^ Center for Clinical and Translational Science The Ohio State University Wexner Medical Center Columbus OH USA

## Abstract

**Objective:**

To test the hypotheses that decomposition electromyography (dEMG) motor unit action potential (MUAP) amplitude and firing rate are altered in SMA; dEMG parameters are associated with strength and function; dEMG parameters are correlated with traditional electrophysiological assessments.

**Methods:**

Ambulatory and non‐ambulatory adults with SMA on nusinersen and healthy controls were enrolled. MUAPs were decomposed from multielectrode surface recordings during 30‐s maximum contraction of the abductor digiti minimi (ADM). Isometric strength, upper limb function, patient‐reported function, and standard electrophysiologic measures of the ADM (compound muscle action potential [CMAP], single motor unit potential [SMUP], motor unit number estimation [MUNE]) were collected.

**Results:**

dEMG MUAP amplitudes were higher in ambulatory versus control and non‐ambulatory groups and were higher in controls versus non‐ambulatory SMA. In contrast, dEMG firing rates were higher in ambulatory versus non‐ambulatory and control groups but similar between non‐ambulatory and control. dEMG parameters showed moderate to strong positive correlation with strength and function whereas CMAP and MUNE better correlated with function than strength. SMUP did not correlate with strength, function, or dEMG MUAP amplitude. dEMG parameters show overall good test–retest reliability.

**Interpretation:**

dEMG provided reliable, noninvasive measure of MUAP amplitude size and firing rate and revealed divergent patterns across disease severity in adults with SMA. Firing rate enhancement, as seen in milder SMA, may provide a therapeutic avenue for improving function in more severe SMA, where firing rates appear preserved. MUAP amplitude size and firing rate, quantified with dEMG, may be promising monitoring biomarker candidates for noninvasive assessment of SMA.

## Introduction

Spinal muscular atrophy (SMA) is an autosomal recessive motoneuronal disorder that occurs at a frequency of about 1 in 11,000 live births.^1^ SMA is caused by the reduction of survival motor neuron (SMN) protein due to homozygous loss of the *SMN1* gene; low levels of full length SMN protein produced by a second similar gene, SMN2, are insufficient for normal motoneuronal function.^2^, ^3^, ^4^ Three SMN‐restoring treatments are currently approved: *SMN1* gene replacement (onasemnogene abeparvovec‐xioi) and small molecule (risdiplam) and antisense oligonucleotide (nusinersen) strategies to increase full length SMN from *SMN2*.^5^, ^6^, ^7^ Despite significant and meaningful impact of these treatments on survival and motor function, the effects of SMN restoration are incompletely understood and vary across individuals. Thus, there is a need for informative and reliable biomarkers to assess disease status and monitor response to treatment.

The classical pathophysiological findings in SMA include spinal motoneuron losses seen postmortem as well as denervation on needle electromyography and muscle biopsy.^8^ Electrophysiological motor unit assessments have demonstrated reductions of motor unit number estimation (MUNE) using a variety of electrophysiological approaches in clinical populations and models of SMA.^9^, ^10^, ^11^, ^12^, ^13^, ^14^, ^15^, ^16^, ^17^ Electrophysiological motor unit losses are accompanied by increases of motor unit size via collateral sprouting, which compensate for motor unit losses for a time but eventually lead to muscle atrophy and functional decline if left untreated.^9^, ^18^, ^19^, ^20^, ^21^, ^22^ However, voluntary muscle function is dependent not only on sufficient motor unit numbers, but also on modulation of motor unit firing rates to fully activate and control muscle contraction.^23^, ^24^, ^25^, ^26^ While traditional electrical stimulation‐evoked electrophysiological measures can identify motor unit losses and compensatory enlargement in SMA, these techniques are unable to quantify voluntary motor unit recruitment or firing.

Newer decomposition electromyography (dEMG) methods use artificial intelligence approaches to discern motor unit potentials from surface‐recorded interference patterns and allow assessment of motor unit firing rates and motor unit action potential size.^27^, ^28^, ^29^, ^30^, ^31^, ^32^, ^33^, ^34^, ^35^, ^36^ To determine the potential usefulness of dEMG in the study of SMA, our objectives were to test the hypotheses that (1) dEMG motor unit action potential amplitude and firing rate are altered in SMA, (2) these dEMG parameters are associated with measures of strength and function, and (3) these dEMG parameters are also correlated with traditional electrophysiological assessments.

## Methods

### Study overview and timeline

This was a prospective study performed in conjunction with a previously published open‐label study to investigate the effects of nusinersen in ambulatory and non‐ambulatory adults with SMA.^10^, ^11^ Inclusion criteria for participants with SMA were ages 18–70 years old, genetically confirmed SMA, and medically stable to tolerate assessments (i.e., effective management of any co‐morbidities and no acute illness or injury). Patients were excluded if they were deemed medically unstable (i.e., acute illness or injury such as pneumonia, urinary tract infection, kidney stones, or fracture that would interfere with assessments), could not provide informed consent, or had a pacemaker/cardiac device. Inclusion criteria for healthy control participants were ages 18–70 years old and have no known neurologic conditions or chronic medical illnesses. Data were analyzed from participants on chronic, stable treatment with nusinersen (10 months or more). A participant was classified as “ambulatory” if they were able to walk 30 feet without assistive devices at the time of enrollment. This study was approved by the Institutional Review Board at The Ohio State University. Written consent was obtained prior to enrollment of all participants; visits were conducted between November 2019 and March 2022.

### Outcome measures

#### Standard ulnar electrophysiology

Maximum ulnar compound muscle action potential (CMAP), average single motor unit potential (SMUP), and multipoint incremental motor unit number estimation (MUNE) were performed on the abductor digiti minimi (ADM) of the dominant limb using a clinical electrodiagnostic system (Natus Medical Inc., Middleton, WI) and methods as previously described.^9^, ^37^ For the CMAP, the G1 primary recording electrode was placed over the motor point of the ADM at the midpoint of the line drawn between the ulnar aspect of the fifth metacarpophalangeal (MCP) joint and the ulnar aspect of the pisiform bone. The G2 reference electrode was placed at the base of the fifth proximal phalanx where it intersects with the MCP joint. An adhesive ground electrode was placed on the back of the hand. Stimulation was applied at the wrist starting with stimulus duration of 50 microseconds and intensity sufficient to elicit a maximum CMAP. To ensure that the motor point has been selected and maximum CMAP amplitude is recorded, the G1 electrode may be moved 2–3 mm distal to the initial placement, then 2–3 mm proximal to the initial placement for a minimum total of three measures.

For MUNE, the ulnar nerve was stimulated at three sites: 2 cm proximal to the wrist crease, 4 cm proximal to the first site, and 1 cm proximal to the ulnar groove at the elbow. Settings included: filter at 10 Hz–10KHz, amplifier at 50 microvolt per division, stimulus control to allow 1/10 milliamp stimulus, stimulus rate at 1/s, and stimulation using self‐adhesive circular motor electrodes. At each of the stimulation sites, the optimal stimulation location was determined by moving the stimulator until the largest response was obtained using submaximal stimulus intensity. An envelope of three incremental all or none superimposed responses of >25 microvolt in amplitude was obtained at each of the stimulation sites; measurement of the negative peak amplitude from baseline was used for calculation. The average SMUP amplitude was obtained by adding the three response amplitudes at each of the three stimulation sites and dividing by nine. The MUNE value was calculated by dividing maximum CMAP amplitude by the average SMUP amplitude.

#### Decomposition electromyography (dEMG)

Surface electromyographic data were collected using the Trigno Avanti Platform and Trigno Galileo Sensor (Delsys Inc., Natick, MA). Skin was cleaned and prepared with alcohol. The wireless sensor recording electrode was placed over the ADM of the participants' dominant hand and the reference electrode was placed on the distal and ventral aspect of the same forearm. Signal quality was tested using a submaximal contraction. Once sufficient signal was confirmed via visual inspection, a single trial of 30 seconds was performed during which the participant was asked to maximally abduct the fingers; verbal cues for effort were provided throughout the trial. Data were processed using NeuroMap Software for automated decomposition of the surface recordings (Delysis Inc., Natick, MA).^27^, ^28^, ^29^, ^30^, ^32^ The Neuromap software reports accuracies and location errors for each motor unit as defined by the decompose synthesize decompose compare method, which uses a physiologically realistic signal synthesized from motor unit action potentials obtained from the decomposition of the real surface EMG (sEMG) signal; the algorithm inspects the signal for clear uncontaminated motor unit shapes, then uses those shapes to find all motor unit firing locations in the sEMG signal, including where motor units are superimposed.^28^, ^31^, ^38^ Parameters of interest include average motor unit action potential (MUAP) amplitude, peak motor unit firing rate (MUFR), and average MUFR.

#### Isometric muscle strength testing

Muscle strength of the elbow flexors and extensors were assessed bilaterally in kilograms using a handheld dynamometer (Citec, Netherlands). These muscles were chosen for analysis as they were the most consistently collectable upper extremity muscle strength data available across the functional spectrum. Ideal positioning for elbow flexors was supine with 90 degrees of elbow flexion, shoulder adducted to side, and forearm in supination. However, for people with forearm contractures, neutral positioning was permitted since this reflects how participants would use elbow flexion strength and movement in their everyday lives. Additionally, for those unable to transfer out of their power wheelchairs, tilt‐in‐space and recline functions were used to obtain supine position. The same positioning was used for elbow extension except with the forearm always in neutral position.^39^ The participant was instructed to perform a 5‐s maximum voluntary isometric contraction (make test) and was given verbal cues for effort. Two trials within 10% were performed and the highest value was recorded. An average of the left and right values was used for analysis. Handheld dynamometry is a valid and reliable measure in people with SMA.^40^, ^41^

#### Revised upper limb module (RULM)

Upper limb function was assessed using the Revised Upper Limb Module (RULM), which has been developed specifically for assessment of the SMA population.^42^ The RULM is a 19‐item outcome measure scored on a 3‐point ordinal scale (0–2) (except one item is scored either 0 or 1), where each score corresponds with a specific level of performance; maximum score is 37 and higher scores reflect better function. Of the 19 items, 8 items require shoulder flexion/abduction to receive any score, 4 items focus on hand dexterity and strength, 5 items require elbow flexion/extension in either anti‐gravity or gravity‐eliminated positions to receive any score, and 2 items use combined movements (i.e., shoulder and elbow or elbow and hand) with the score depending on extent of task completion. This test has shown validity and reliability in adults with SMA across the functional spectrum.^42^, ^43^, ^44^

#### Revised SMA functional rating scale (SMAFRS)

The Revised SMA Functional rating scale (SMAFRS) is a patient‐reported outcome measure administered by a trained evaluator. SMAFRS captures the level of assistance required with common daily activities including eating, grooming, toileting, bathing, dressing, turning in bed, transfers, walking, climbing stairs, and respiratory status. There are 10 items scored on a 5‐point ordinal scale (0–4) where higher scores indicate greater independence; maximum score is 40. It has shown good test–retest reliability as well as correlation with other measures of physical function in adults with SMA.^45^

### Statistical analysis

Statistical analyses were performed using GraphPad Prism version 8.4.3 (GraphPad Software, San Diego, CA). Descriptive statistics of participant demographics and characteristics were calculated. One‐way ANOVA analyses with Tukey Multiple Comparison post hoc were used to determine group differences by functional status. Reported *P*‐values are the adjusted for multiple comparisons. Based on results of assumption testing, either Pearson or Spearman correlations were used to determine associations among dEMG measures (average MUAP amplitude, peak MUFR, and average MUFR), traditional electrophysiology measures (MUNE, CMAP, and SMUP), and measures of strength and function (elbow flexion, elbow extension, RULM, and SMAFRS). There were no corrections made for multiple comparisons for the correlation analyses due to the high risk for Type II error with the large number of correlations being performed; instead, we looked for trends in the data to suggest whether a Type I error may have occurred (i.e., low number of seemingly sporadic yet significant correlations). Group difference analyses used individual motor unit data (i.e., multiple motor units could be contributed by one participant) while correlation analyses used average motor unit data (i.e., mean values per participant). Alpha was set to *P* < 0.05. Two additional analyses were completed for dEMG measures using IBM SPSS Statistics 29.0.1.0 (IBM, Armonk, NY) and average motor unit data. Intraclass correlation coefficient (ICC) estimates and their 95% confidence intervals (95% CI) were calculated based on a mean‐measurement (*k* = 2), absolute‐agreement, two‐way mixed‐effects model.^46^ K‐means cluster analysis was performed using Euclidean Distance similarity measure, and two clusters were chosen to parallel the standard dichotomous ambulatory status clinical classification.^47^, ^48^

## Results

### Characteristics of study participants

Demographics and clinical characteristics of the cohort are presented in Table 1. Decomposition EMG, strength, and functional data from 28 adult participants with SMA were analyzed. Five participants scored the maximum value on the RULM, and no participants scored the maximum value on the SMAFRS. Decomposition EMG data were also collected from 8 control participants (4 male/4 female, aged 39.63 ± 11.35 years [ranging 26–61 years old]). Control and SMA groups were balanced for age and sex. Two‐hundred seventy‐five motor units were included in analysis for the SMA cohort (ambulatory *n* = 103, non‐ambulatory *n* = 172) and 101 motor units were included in the control cohort. A participant's motor units were only included if they had a minimum of 2 motor units decomposed at greater than 85% accuracy. On average, 10 motor units were detected per SMA participant (range 2–19) and 13 per control participant (range 6–20). Traditional electrophysiology measures (SMUP, CMAP, MUNE) were only available for 11 of the 28 participants (5 ambulatory, 6 non‐ambulatory) due to a combination of equipment malfunction and technical difficulties with administration. The average time on nusinersen was 28.5 months (36 months for ambulatory participants and 23.7 months for non‐ambulatory); only 2 participants (both non‐ambulatory) had been on nusinersen for 10 months.

**Table 1:** Demographic and clinical characteristics.

	SMA (*n* = 28)	Ambulatory (*n* = 11)	Non‐ambulatory (*n* = 17)
Age (years)	38.96 ± 11.53 [20–60]	38.82 ± 3.49 [20–60]	39.06 ± 2.88 [23–60]
Age of symptom onset (years)	3.268 ± 3.334 [0.5–17]	5.32 ± 1.29 [2–17]	1.94 ± 0.39 [0.5–5.5]
Disease duration (years)	35.7 ± 11.27 [17–56.75]	33.5 ± 3.63 [17–55]	37.12 ± 2.64 [22–56.75]
SMA type	Type 2: 9 (32%) / Type 3a: 6 (21%) / Type 3b: 13 (47%)	Type 3b: 11 (100%)	Type 2: 9 (53%) / Type 3a: 6 (35%) / Type 3b: 2 (12%)
Sex	12M/16F	6M/5F	6M/11F
Race	Caucasian: 27 (96%)	Caucasian: 10 (91%) Black or African‐American: 1 (9%)	Caucasian: 17 (100%)
*SMN 2* Copy Number	3 copies: 19 (68%) / 4 copies: 9 (32%)	3 copies: 4 (36%) / 4 copies: 7 (64%)	3 copies: 15 (88%) / 4 copies: 2 (12%)
Scoliosis (*n*/% yes)	20 (71%)	5 (45%)	15 (88%)
Scoliosis surgery (*n*/% yes)	10 (36%)	0 (0%)	10 (59%)
Uses noninvasive ventilatory pressure support (*n*/% yes)	8 (29%)	1 (9%)	7 (41%)
Uses enteral nutrition (*n*/% yes)	2 (7%)	0 (0%)	2 (12%)
Uses leg bracing (*n*/% yes)	0 (0%)	0 (0%)	0 (0%)
Uses manual wheelchair (*n*/% yes)	2 (7%)	1 (9%)	1 (6%)
Uses walker (*n*/% yes)	1 (4%)	1 (9%)	0 (0%)
Uses power mobility (*n*/% yes)	Power wheelchair: 19 (67%) Scooter: 1 (4%)	4 (36%)	16 (94%)
Revised Upper Limb Module score	21.86 ± 12.93 [0–37]	33.55 ± 3.698 [28–37]	14.29 ± 10.91 [0–36]
SMA Functional Rating Scale score	18.57 ± 12.88 [2–38]	32.27 ± 3.797 [25–38]	9.706 ± 7.630 [2–27]

	SMA (*n* = 11)	Ambulatory (*n* = 5)	Non‐Ambulatory (*n* = 6)
Compound muscle action potential (mV)	7.036 ± 2.921 [1.40–11.20]	8.920 ± 2.039 [7.30–11.20]	5.467 ± 2.689 [1.40–8.20
Single motor unit potential (mV)	0.0985 ± 0.0341 [0.070–0.19]	0.0888 ± 0.0201 [0.070–0.12]	0.1065 ± 0.0428 [0.075–0.190]
Motor unit number estimation	77.64 ± 38.81 [18–158]	103.4 ± 31.94 [77–158]	56.17 ± 31.26 [18–89]

Ages, disease duration, and functional outcomes reported as mean and standard deviation followed by min and max values. All participants with SMA had been treated with nusinersen for an average of 28.5 months (range 10–48 months).

### Motor unit action potential amplitudes and firing rates are altered across severities of SMA and compared with controls

**Figure 1:**
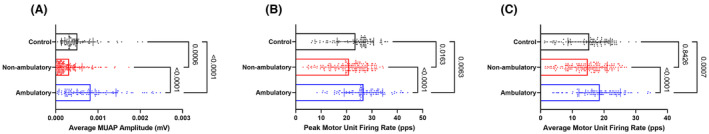
Ordinary one‐way ANOVA was used to analyze comparisons of average motor unit action potential (MUAP) amplitude (A), peak motor unit firing rate (B), and average motor unit firing rate (C) in individual motor units among ambulatory (blue) and non‐ambulatory (brown) adults with SMA and controls (black).

Average MUAP amplitude, peak MUFRs, and average MUFRs were compared across three groups (Fig. 1) including healthy controls (8 participants, 101 MU), ambulatory participants with SMA (11 participants, 103 MU), and non‐ambulatory participants with SMA (17 participants, 172 MU). There are significant differences in the average MUAP amplitude (Fig. 1A) and peak MUFR (Fig. 1B) of ambulatory participants compared to non‐ambulatory (*P* < 0.0001 for both) and controls (*P* < 0.0001, *P* = 0.0063, respectively) as well as a significant difference between non‐ambulatory participants compared to controls (*P* = 0.0006, *P* = 0.0163, respectively). The average MUAP amplitude and peak MUFR of ambulatory participants were higher than control participants whose values were higher than non‐ambulatory participants. As shown in Figure 1C, there is a significant difference in average MUFR of ambulatory participants compared to non‐ambulatory (*P* < 0.0001) and controls (*P* = 0.0007), while there is no difference between non‐ambulatory and controls. The average MUFR of ambulatory participants was higher than control and non‐ambulatory participants. Descriptive statistics of individual motor unit characteristics are reported in Table 2.

**Table 2:** Descriptive statistics of individual motor unit characteristics.

	SMA (*n* = 275)	Ambulatory (*n* = 103)	Non‐ambulatory (*n* = 172)	Controls (*n* = 101)
Average MUAP amplitude (mV)	0.0005050 ± 0.0005054 [0.00001632–0.002476]	0.0008248 ± 0.0006004 [0.00003392–0.002476]	0.0003136 ± 0.0003094 [0.00001632–0.001733]	0.0005122 ± 0.0003724 [0.00004571–0.002051]
Peak motor unit firing rate (pps)	23.09 ± 7.835 [2.886–44.09]	26.65 ± 7.594 [8.365–44.09]	20.97 ± 7.197 [2.886–34.45]	23.50 ± 7.178 [6.190–36.18]
Average motor unit firing rate (pps)	16.28 ± 6.564 [1.442–34.61]	18.65 ± 6.548 [3.834–34.61]	14.86 ± 6.169 [1.442–27.07]	15.31 ± 6.712 [1.245–30.09]

Amplitude and firing rates are reported as mean and standard deviation followed by min and max values.

### Motor unit action potential amplitudes and firing rates are associated with measures of muscle strength and physical function

**Figure 2:**
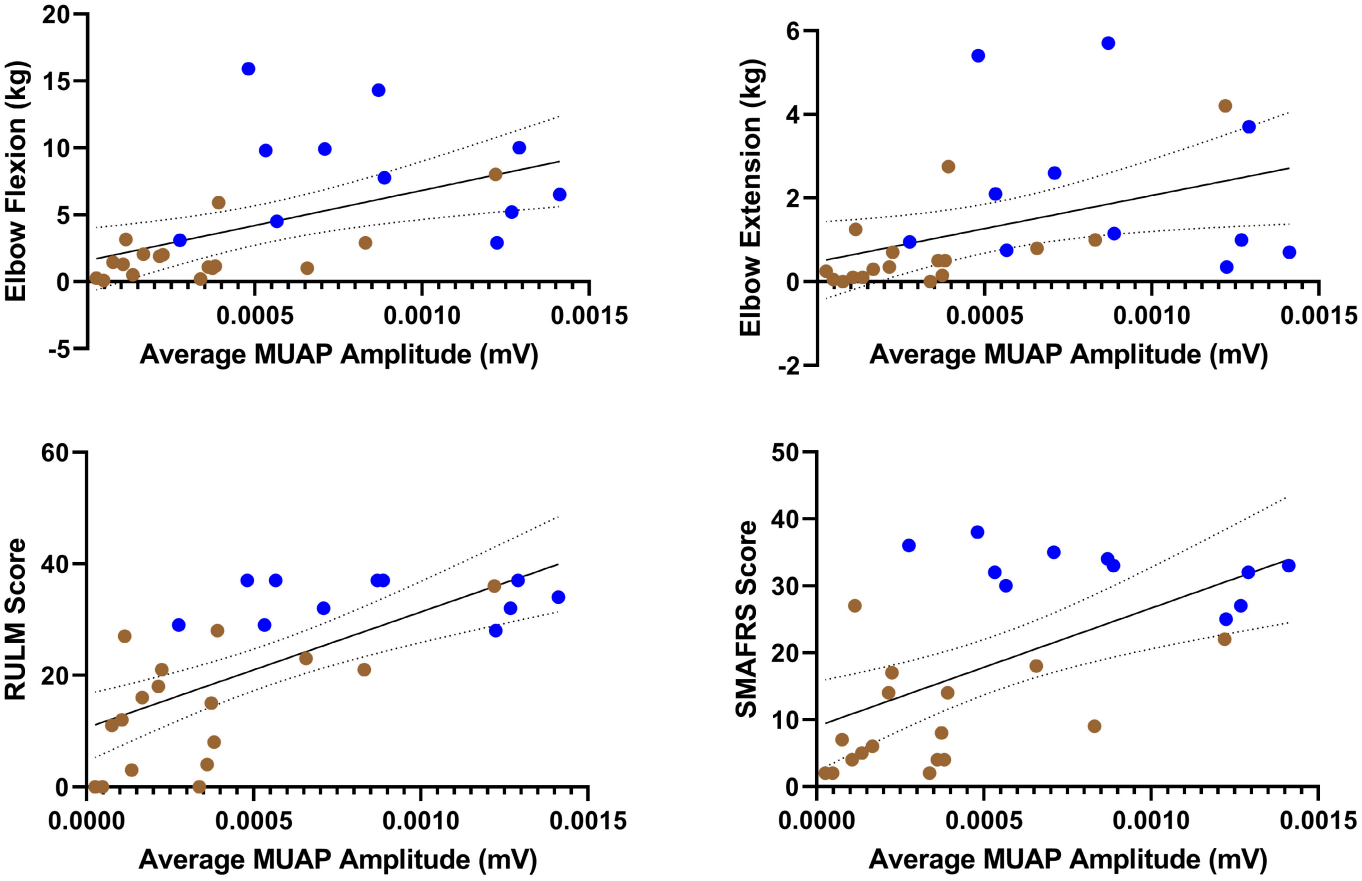
Correlations of average motor unit action potential (MUAP) amplitude compared to measures of strength and function in ambulatory (blue) and non‐ambulatory (brown) adults with SMA (*n* = 28).

**Figure 3:**
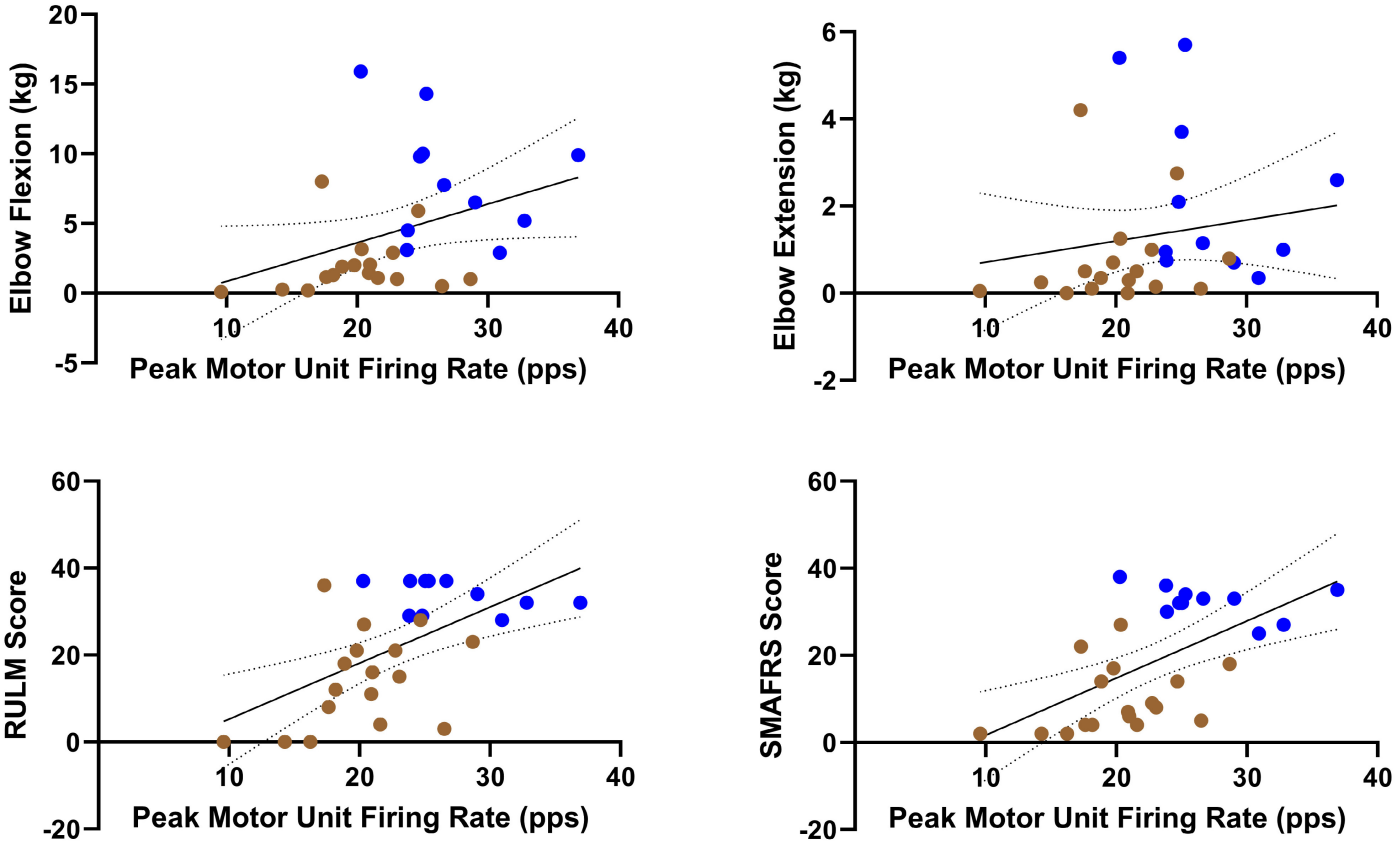
Correlations of peak motor unit firing rate compared to measures of strength and function in ambulatory (blue) and non‐ambulatory (brown) adults with SMA (*n* = 28).

**Figure 4:**
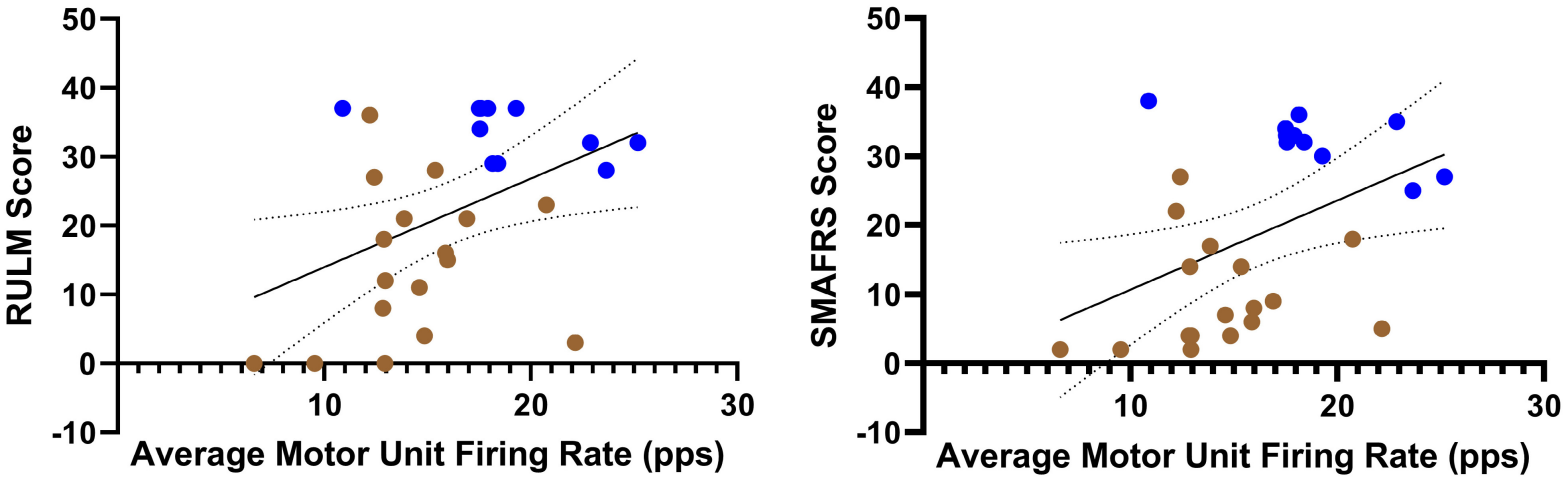
Correlations of average motor unit firing rate compared to measures of function in ambulatory (blue) and non‐ambulatory (brown) adults with SMA (*n* = 28).

Figures 2, 3, 4 shows the three dEMG parameters (average MUAP amplitude, peak MUFR, and average MUFR) in correlation with measures of function (RULM and SMAFRS), and measures of strength (elbow flexion and extension) for ambulatory and non‐ambulatory participants. As shown in Figure 2, average MUAP amplitude shows moderate to strong, significant correlations with RULM (Pearson *r* = 0.6887, *P* < 0.0001), SMAFRS (Pearson *r* = 0.5883, *P* = 0.0010), elbow flexion strength (Spearman *r* = 0.6663, *P* = 0.0001), and elbow extension strength (Spearman *r* = 0.6341, *P* = 0.0003). As shown in Figure 3, peak MUFR shows moderate, significant correlations with RULM (Pearson *r* = 0.5758, *P* = 0.0013), SMAFRS (Pearson *r* = 0.5888, *P* = 0.0010), and elbow flexion strength (Spearman *r* = 0.4684, *P* = 0.0119), while peak MUFR shows low but significant correlation with elbow extension strength (Spearman *r* = 0.3821, *P* = 0.0448). As shown in Figure 4, average MUFR shows moderate, significant correlations with RULM (Pearson *r* = 0.4332, *P* = 0.0213), and SMAFRS (Pearson *r* = 0.4375, *P* = 0.0199). This moderate correlation is weaker than seen in peak MUFR.

### Evoked single motor unit potential does not correlate with measures of strength and function

**Figure 5:**
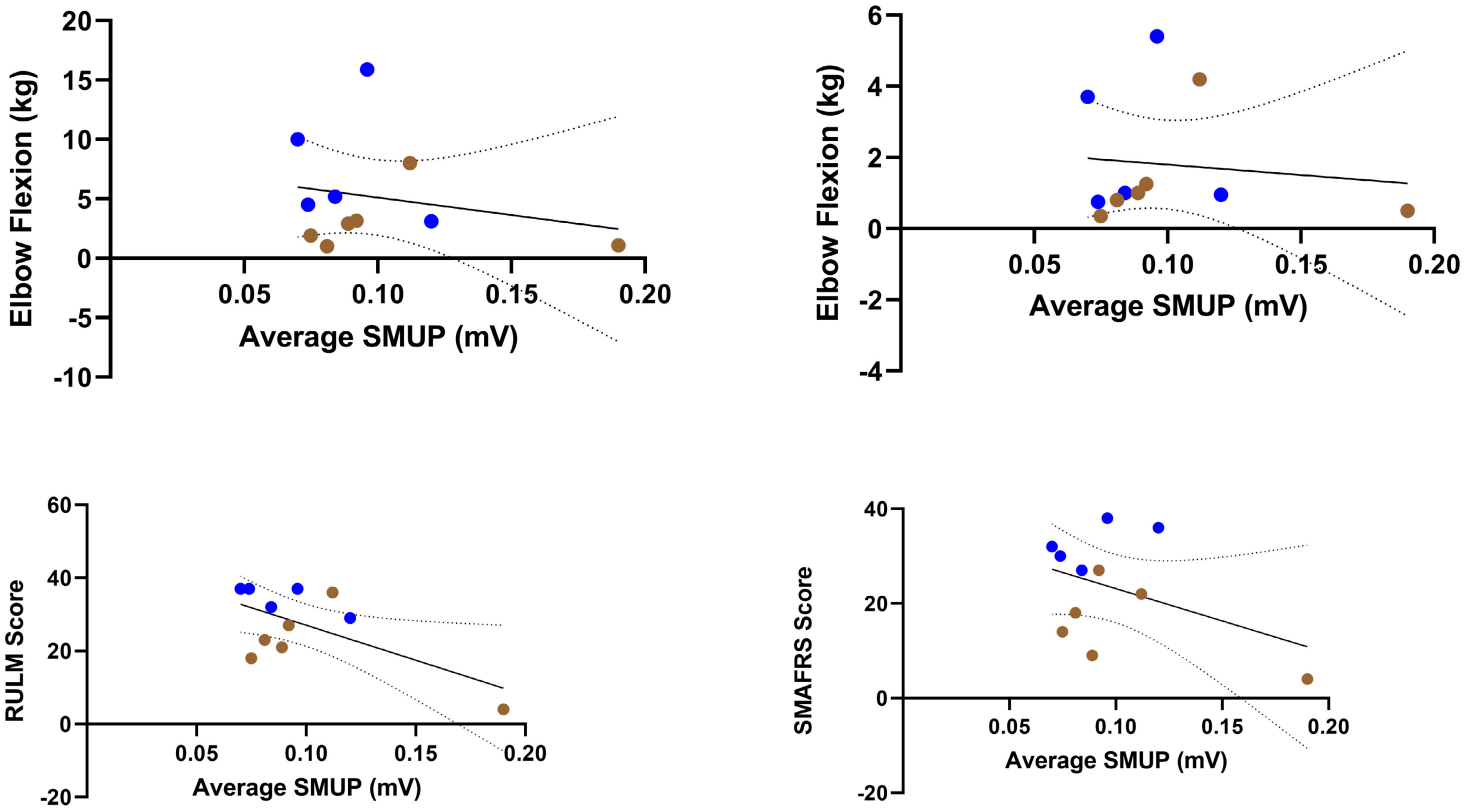
Correlations of average single motor unit potential (SMUP) compared to measures of strength and function in ambulatory (blue) and non‐ambulatory (brown) adults with SMA (*n* = 11).

**Figure 6:**
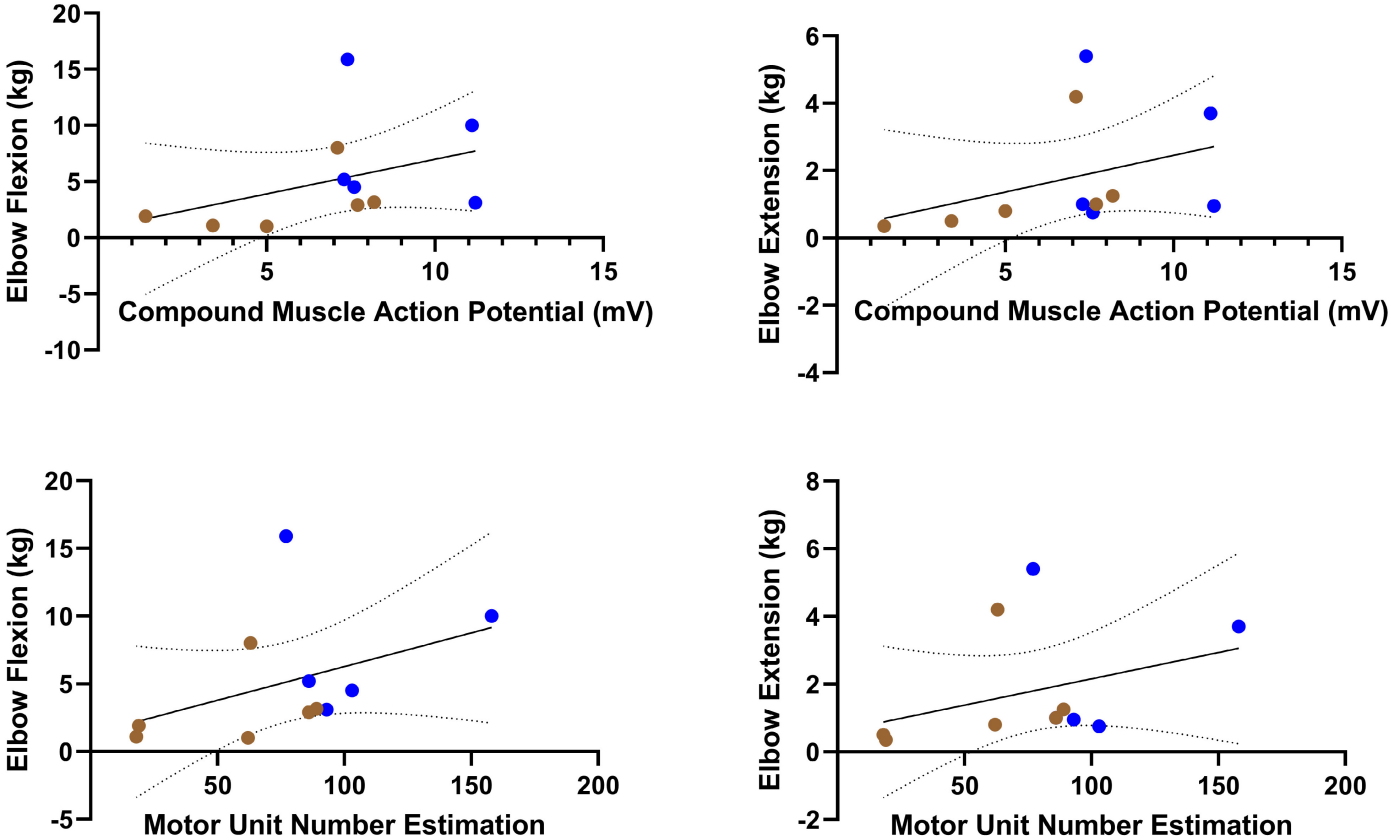
Correlations of compound muscle action potential (CMAP) and motor unit number estimation (MUNE) compared to elbow flexion and elbow extension strength in ambulatory (blue) and non‐ambulatory (brown) adults with SMA (*n* = 11). Please note that there are two completely superimposed data points in the figure on the right (MUNE = 86, elbow extension = 1.0 kg); one was non‐ambulatory and one was ambulatory.

**Figure 7:**
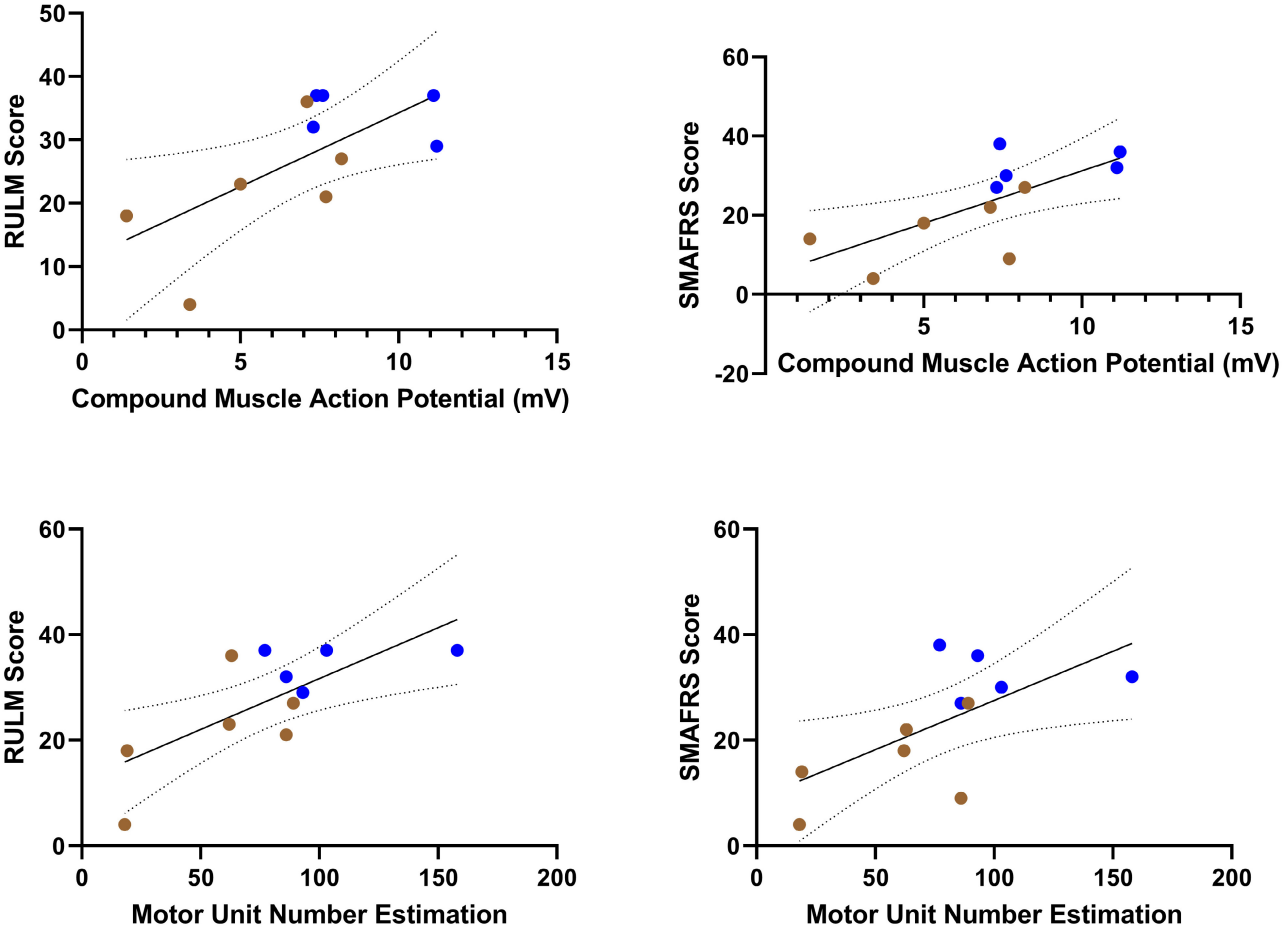
Correlations of compound muscle action potential (CMAP) and motor unit number estimation (MUNE) compared to Revised Upper Limb Module (RULM) and SMA Functional Rating Scale (SMAFRS) in ambulatory (blue) and non‐ambulatory (brown) adults with SMA (*n* = 11).

Average SMUP shows very low to low, insignificant correlations with each measure of strength and function: elbow flexion (Spearman *r* = −0.1091, *P* = 0.7545), elbow extension (Spearman *r* = 0.1412, *P* = 0.6783), RULM (Spearman *r* = −0.3028, *P* = 0.3654), and SMAFRS (Spearman *r* = −0.1093, *P* = 0.7488). This is illustrated in Figure 5. Additionally, CMAP and MUNE exhibited low to moderate, insignificant correlations with elbow flexion strength (Spearman *r* = 0.4182, *P* = 0.2030; Spearman *r* = 0.5194, *P* = 0.1044, respectively) and elbow extension strength (Spearman *r* = 0.4510, *P* = 0.1651; Spearman *r* = 0.3425, *P* = 0.2996, respectively) (Fig. 6). In contrast, CMAP and MUNE show strong, significant correlations with RULM (Pearson *r* = 0.6574, *P* = 0.0279; Pearson *r* = 0.7231, *P* = 0.0119, respectively) and SMAFRS (Pearson *r* = 0.7024, *P* = 0.0160; Pearson *r* = 0.6551, *P* = 0.0287, respectively) (Fig. 7).

### Average motor unit action potential amplitude does not correlate with evoked single motor unit potential

**Figure 8:**
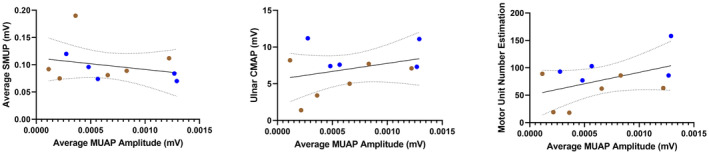
Correlations of average motor unit action potential (MUAP) amplitude compared to traditional electrophysiology measures in ambulatory (blue) and non‐ambulatory (brown) adults with SMA (*n* = 11). CMAP, compound muscle action potential; SMUP, single motor unit potential.

**Figure 9:**
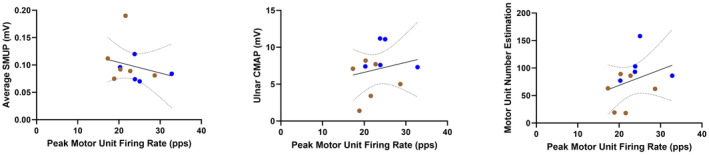
Correlations of peak motor unit firing rate compared to traditional electrophysiology measures in ambulatory (blue) and non‐ambulatory (brown) adults with SMA (*n* = 11). CMAP, compound muscle action potential; SMUP, single motor unit potential.

Although they are both considered indices of motor unit collateral sprouting and remodeling, average MUAP amplitude showed only weak and insignificant correlation with SMUP (Spearman *r* = −0.3545; *P* = 0.2862) (Fig. 8). Weak to moderate and insignificant correlation was also seen when comparing average MUAP amplitude to CMAP and MUNE (Pearson *r* = 0.3250, *P* = 0.3295; Pearson *r* = 0.4667, *P* = 0.1479, respectively) (Fig. 8). As shown in Figure 9, SMUP, CMAP, and MUNE show a similar low to moderate and insignificant correlation with peak MUFR (Spearman *r* = −0.4364, *P* = 0.1826; Pearson *r* = 0.2071, *P* = 0.5413; Pearson *r* = 0.3295, *P* = 0.3225, respectively); this same pattern holds for correlation of average MUFR with SMUP, CMAP, and MUNE (Spearman *r* = −0.418, *P* = 0.203; Pearson *r* = 0.2058, *P* = 0.5437; Pearson *r* = 0.3109, *P* = 0.3522, respectively) (not pictured).

### ICC values for dEMG parameters indicate overall good test–retest reliability

Repeated measures were available for a subset of our study population (*n* = 14). Research visit interruptions due to the COVID pandemic limited repeated measure collection for all 28 participants. This data was used to determine test–retest reliability. Time between measurements averaged 11.86 ± 5.35 months and ranged 4–22 months. Average MUAP amplitude ICC was .891, 95% CI [0.581, 0.967]. Peak MUFR ICC was .881, 95% CI [0.623, 0.962]. Average MUFR ICC was .824, 95% CI [0.446, 0.944].

### K‐means cluster analysis demonstrates dEMG parameter cluster agreement with ambulatory status clinical classification

K‐means cluster analysis was used to group each dEMG parameter into 2 clusters in an unbiased way. We then compared the clusters to standard clinical classification to see how well they agreed. Table 3 shows the results of the K‐means cluster analysis for average MUAP amplitude, peak MUFR, and average MUFR. “Low Cluster” and “High Cluster” refer to the relative grouping of dEMG values, which would have been expected to identify worse (i.e., non‐ambulatory) and better functioning (i.e., ambulatory) participants, respectively. For average MUAP amplitude, agreement between cluster placement and ambulatory status overall occurred 75% of the time, although more frequently for non‐ambulatory (88%) than ambulatory (55%) participants. For peak and average MUFR, agreement overall occurred 86% of the time with 91% agreement for ambulatory and 82% agreement for non‐ambulatory participants.

**Table 3:** Results of the K‐means cluster analysis.

	Low cluster	High cluster	% Agreement
Average MUAP amplitude			75%
Ambulatory (*n* = 11)	5	6	55%
Non‐ambulatory (*n* = 17)	15	2	88%
Peak MUFR			86%
Ambulatory	1	10	91%
Non‐ambulatory	14	3	82%
Average MUFR			86%
Ambulatory	1	10	91%
Non‐ambulatory	14	3	82%

“Low” and “High” refer to the relative grouping of amplitude and firing rate values where Low would be expected to have worse function (i.e., Non‐ambulatory) and High would be expected to have better function (i.e., ambulatory). Percent agreement refers to the proportion of participants whose ambulatory status agreed with expected cluster placement.

## Discussion

In the era of SMN‐restoring therapies, it is increasingly imperative to have patient‐centered methods to monitor responses to therapies and assess disease status and progression across the functional spectrum.^49^, ^50^ In this study, we demonstrated that dEMG detected differences in MUAP amplitude size and firing rate between SMA patients and healthy controls and across phenotypic severities in adults with SMA. Furthermore, dEMG measures correlated with strength and functional measures, suggesting a positive relationship between extent of motor unit compensation and motor performance. dEMG also provides information about motor unit activation while sparing patients the potential discomfort of traditional electrophysiologic testing (i.e., nerve stimulation). Together these findings position dEMG as a compelling candidate biomarker for patients with SMA.

### Motor units demonstrate compensatory increases of MUAP amplitude size and firing rate in ambulatory adults with SMA

Motor unit loss and muscle denervation are well‐established in SMA.^8^ Following motor unit losses, the remaining motor units undergo collateral sprouting and reinnervation to maintain function.^9^, ^18^ Yet, natural history studies of SMA have shown progressive loss of enlarged motor units with age and decline in functional status.^9^, ^18^, ^22^ For adults with SMA, treatment with nusinersen appears to stabilize disease progression, and higher functioning patients may even see modest improvements.^10^, ^11^, ^51^, ^52^, ^53^, ^54^, ^55^, ^56^ While our study did not investigate impact of nusinersen, our results suggest potential mechanisms that could be contributing to this alternate trajectory. MUAP amplitudes are the summation of muscle fiber action potential amplitudes, and MUAP amplitude size can be related to the number of muscle fibers, size of muscle fibers, and extent of innervation. For ambulatory participants, significantly higher average MUAP amplitudes on dEMG could indicate preservation of large motor units, presence of enlarged motor units (units with collateral sprouting), maintenance of muscle fiber size, or hypertrophy of muscle fibers. This is consistent with previous findings of both untreated and treated adults classified as either ambulatory or Type 3/4 as well as preclinical treatment studies and regardless of method used to quantify this phenomenon (i.e., SMUP, MUSIX, MScanFIt, direct visualization).^11^, ^19^, ^20^, ^57^, ^58^

In contrast to motor unit enlargement, the change of motor unit firing rates during neuromuscular pathologies has received less attention. Our findings of higher motor unit firing rates in the ambulatory participants may indicate a compensatory enhancement for motor activation. Contrasting findings of reduced firing rates have been noted in chronic inflammatory demyelinating polyradiculoneuropathy, an autoimmune peripheral neuropathy.^59^ In ALS, a more selective motor neuron disorder more akin to SMA, increases in motor unit firing rates compared to controls have been seen, similar to the findings in our study.^60^ Additionally, higher firing rates correlated with higher levels of function in patients with ALS.^60^ Together with our findings, this suggests that firing rate increases may play a compensatory role in maintaining the function of adults with motor neuron diseases.

### MUAP amplitude size is reduced and firing rate is unchanged in non‐ambulatory adults with SMA

The current study demonstrated that more severely affected, non‐ambulatory adults show reduced MUAP amplitude size but minimal change of firing rates compared to controls, contrasting observations in ambulatory patients with SMA. The presence of significantly smaller average MUAP amplitudes may indicate losses of large motor units, retraction of enlarged, collateral sprouted motor units, or muscle fiber atrophy, all of which could be expected in more severe SMA. However, firing rates of the remaining motor units in non‐ambulatory adults appear relatively preserved. This is consistent with previous findings that used a similar dEMG method in children with Type II SMA.^61^ Given this preservation, targeted approaches to enhance motor unit firing rates may be an effective therapeutic strategy for improving function in more severe SMA. Firing rates have been shown to increase with augmentation of persistent inward currents (PICs), which are ongoing depolarizing currents produced by voltage‐sensitive sodium and calcium channels predominantly on motor neuron dendrites.^62^, ^63^ In humans, augmentation of PICs has been achieved through exercise training, while pre‐clinical studies have demonstrated similar effects by introducing serotonin and noradrenalin.^62^, ^63^, ^64^, ^65^ Investigation into these approaches for SMA may be warranted.

### Decomposition EMG: A potential biomarker in SMA?

A biomarker is “a defined characteristic that is measured as an indicator of normal biological processes, pathogenic processes or responses to an exposure or intervention” and can be subtyped into seven applications: diagnostic, monitoring, response, predictive, prognostic, safety, and susceptibility/risk.^66^ In SMA, dEMG could be a powerful monitoring biomarker used to measure motor unit biologic processes to assess disease status.^66^ Average MUAP amplitude and peak firing rate on dEMG both showed ability to distinguish disease severity, clustering agreement with clinical classification, moderate to strong correlation with measures of physical function, and acceptable test–retest reliability. Moreover, compared to monitoring by clinical outcome measures (COMs) and traditional electrophysiologic biomarkers, dEMG has several advantages.

There are few COMs that are appropriate to administer across the functional spectrum of SMA. Typically administered SMA outcomes measures have significant floor and ceiling effects, thus limiting applicability in individuals with more and less advanced disease.^67^, ^68^, ^69^, ^70^ Furthermore, current measures struggle to capture all developmental stages, with few existing for adults; it is also necessary to switch outcome measures depending on age, leading to discontinuity of assessment.^67^, ^71^, ^72^, ^73^ As children can begin to follow multistep directions around the age of 2, dEMG would provide a method that could be consistently applied throughout the lifespan starting at a very young age and would be independent of developmental stage and functional status.^74^ dEMG is also less physically demanding than most performance‐based COMs and could be administered even in cases when patients are too fatigued to complete a 20–30 item test or have an injury to their leg or shoulder that would also preclude testing.

Evoked single motor unit potential (SMUP) amplitude was anticipated to be the most closely mirroring measure to dEMG average MUAP amplitude, both capturing characteristics of individual motor unit size. However, SMUP and dEMG average MUAP amplitudes showed insignificant correlation and only dEMG showed correlation with measures of physical function. The lack of correlation between SMUP and physical function in SMA is consistent with a prior report.^75^ One possible explanation for these results could be related to the fact that motor unit recruitment thresholds, which dictate recruitment order during voluntary contractions, are bypassed during evoked stimulation. Instead of capturing the natural physiologic functioning of a motor unit, the maximal capacities are being elicited. However, maximal capacity may not reflect what is under volitional control and thus able to contribute to voluntary function.^31^, ^76^, ^77^, ^78^

Finally, dEMG has the benefit of being an extremely well‐tolerated procedure that is also relatively easy to administer. In contrast to traditional electrophysiology, it requires no stimulation or invasive needle insertion and can be administered after brief evaluator instruction. Pain is a common report during nerve conduction studies and needle EMG, which can impact the quality, utility, and experience of these procedures.^79^, ^80^ This is an even more important consideration in the pediatric population, who is even less likely to tolerate discomfort.^81^ Furthermore, technical challenges can reduce the yield of successful procedures; for adults with SMA, this may be more predominant in those with greater disease severity.^10^, ^80^, ^81^, ^82^

### Limitations and future directions

There are several limitations that should be noted with regards to interpreting the generalizability of our findings. First, we only tested one distal hand muscle at a maximal voluntary contraction. As a follow‐up, additional muscles with varied levels of sparing (i.e., proximal and distal, upper and lower extremity, dominant and non‐dominant side) and at different intensities of voluntary contraction (minimum to maximum) should be measured to determine the consistency of our results and whether assessment of multiple muscles improves biomarker utility. Second, the strength and electrophysiologic data were collected from different muscles. While the selection of muscles was based upon standard practices in SMA, it is unclear whether testing of concordant muscles would change the relationships found. Third, due to technical difficulties, the traditional electrophysiology sample size is relatively small, which could have impacted the statistical power. A prospectively powered study that also includes additional alternative electrophysiology methodologies such as MScanFIt would be valuable in determining the relative strengths of the dEMG approach for motor unit assessment. Fourth, the floor and ceiling effects of the RULM could have influenced correlation findings with this measure; however, this seems unlikely given similar correlation findings with the SMAFRS, which did not experience floor or ceiling effects. Finally, dEMG may be useful as other types of biomarkers, such as prognostic, predictive, and response biomarkers, but require longitudinal and interventional study designs to test their validity.

### Conclusions

In conclusion, dEMG is a compelling candidate as a biomarker for patients in SMA. It boasts technical ease of administration, high patient tolerability, and ability for consistent use throughout the lifespan, all of which make it both user‐ and patient‐friendly. Furthermore, its correlation with measures of physical function, ability to distinguish between disease severities, and capturing of natural, real‐world motor unit physiologic functioning suggest better overall utility than evoked measurement of individual motor units for assessing disease status and progression. Finally, our results suggest that rescue and/or stabilization of motor units as well as enhanced motor unit firing rates are potential targets for intervention; however, lower functioning individuals potentially have more ability to modulate motor unit firing rates as opposed to undergoing motor unit rescue, which could affect the extent of response to respectively targeted treatments. Future work should explore these different treatment avenues with the goal of maximizing the outcomes for all people with SMA throughout the lifespan.

## Conflict of Interest

KMK: Advisory board for Biogen, member of Neuromuscular Study Group Planning Committee, travel support from Neuromuscular Study Group; SH: salary support from Muscular Dystrophy Association; SJK: consulting for Novartis; BE: research funding from Biogen, Genentech, Alexion, Pharnext, Viela Bio, advisory board for Biogen, Genentech, Argenx, Honoraria from Muscular Dystrophy Association, Stanford University, Travel Support from Cure SMA, Muscular Dystrophy Association, Committee Member for Cure SMA, receipt of drug for clinical trial from Celgene; WDA: research funding from NMD Pharma, Avidity Biosciences, Consulting for NMD Pharma, Avidity Biosciences, Dyne Therapeutics, Novartis, La Hoffman Roche, Genentech, Design Therapeutics, Cadent Therapeutics, Catalyst Pharmaceuticals, Honoraria from University of Rochester, Travel Support from NMD Pharma, Chair/Member of Novartis Data Safety and Monitoring Board, Chair of Neuromuscular Study Group Planning Committee, Patent for “Methods and compositions for treating disorders and diseases using Survival Motor Neuron (SMN) protein.”

## Funding Information

This study was funded by Cure SMA.

## Author Contributions

Conceptualization: Kristina M. Kelly, Amy Bartlett, Stephen J. Kolb, Bakri Elsheikh, and W. David Arnold; formal analysis: Kristina M. Kelly, Jordan Mizell, Ladan Bigdeli, Samuel Paul, Bakri Elsheikh, and W. David Arnold; investigation: Kristina M. Kelly, Marco A. Tellez, Amy Bartlett, Sarah Heintzman, Jerold E. Reynolds, Gary Brent Sterling, Kiran F. Rajneesh, Stephen J. Kolb, Bakri Elsheikh, and W. David Arnold; writing—original draft: Kristina M. Kelly, Jordan Mizell, Ladan Bigdeli, Samuel Paul, Bakri Elsheikh, and W. David Arnold; writing—review and editing: Kristina M. Kelly, Jordan Mizell, Ladan Bigdeli, Samuel Paul, Marco A. Tellez, Amy Bartlett, Sarah Heintzman, Jerold E. Reynolds, Gary Brent Sterling, Kiran F. Rajneesh, Stephen J. Kolb, Bakri Elsheikh, and W. David Arnold; funding acquisition: Stephen J. Kolb, Bakri Elsheikh, and W. David Arnold.
